# Site-Specific Insertion Polymorphism of the MITE *Alex-1* in the Genus *Coffea* Suggests Interspecific Gene Flow

**DOI:** 10.4061/2011/358412

**Published:** 2011-09-27

**Authors:** Christine Dubreuil-Tranchant, Romain Guyot, Amira Guellim, Caroline Duret, Marion de la Mare, Norosoa Razafinarivo, Valérie Poncet, Serge Hamon, Perla Hamon, Alexandre de Kochko

**Affiliations:** ^1^IRD, UMR DIADE, Centre IRD de Montpellier, BP 64501, 34394 Montpellier Cedex 5, France; ^2^UMR ECOFOG INRA, BP 709, 97387 Kourou Cedex, Guyane Française, France; ^3^FOFIFA, BP 1444, Ambatobe, Antananarivo 101, Madagascar

## Abstract

Miniature Inverted-repeat Transposable Elements (MITEs) are small nonautonomous class-II transposable elements distributed throughout eukaryotic genomes. We identified a novel family of MITEs (named *Alex*) in the *Coffea canephora* genome often associated with expressed sequences. The *Alex-1* element is inserted in an intron of a gene at the *CcEIN4* locus. Its mobility was demonstrated by sequencing the insertion site in *C. canephora* accessions and *Coffea* species. Analysis of the insertion polymorphism of *Alex-1* at this locus in *Coffea* species and in *C. canephora* showed that there was no relationship between the geographical distribution of the species, their phylogenetic relationships, and insertion polymorphism. The intraspecific distribution of *C. canephora* revealed an original situation within the E diversity group. These results suggest possibly greater gene flow between species than previously thought. This MITE family will enable the study of the *C. canephora* genome evolution, phylogenetic relationships, and possible gene flows within the *Coffea* genus.

## 1. Introduction

Recently, a new type of molecular marker based on the insertion polymorphism of transposable elements (TEs) was shown to be particularly effective for plant diversity studies [1–4]. Because of their repeated nature and, in some cases, their great number [5, 6], these mobile genetic elements may be inserted at different loci in the genome where they lead to mutations or chromosomal rearrangements. Their activity is responsible for considerable natural polymorphism that can be used to study within and between species diversity and to identify possible population genetic structure and phylogenetic relationships [7, 8].

Among these mobile genetic elements, MITEs (Miniature Inverted-repeat Transposable Elements) form a particular group. MITEs are short (<600 bp) nonautonomous type II transposable elements that are often quite widely distributed in eukaryote genomes but at the same time are highly conserved, within a genome, in size and sequence, indicating that they might originate from a limited number of progenitors [9]. Their even distribution throughout plant genomes makes them an ideal tool for the study of genome evolution and genetic relationships [10–12].

**Figure 1:**
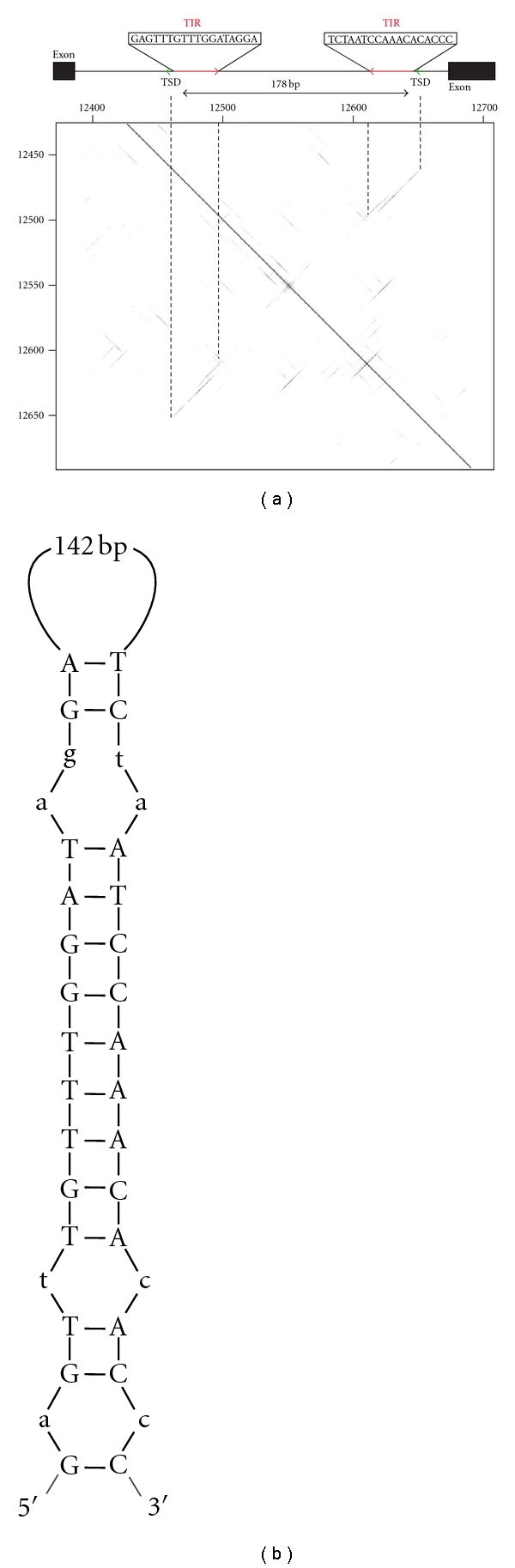
Structural characterization of the *Alex-1* MITE. (a) Dot plot of the MITE against itself allowed the identification of the 18 bp terminal inverted repeats (in red). (b) Folding of *Alex-1* revealed the typical stem loop structure of MITE elements.

Such elements could help to solve problematic phylogenetic relationships among species including those in the genus *Coffea*, which comprises 103 species originating from Africa, Madagascar, and several islands in the Indian Ocean [13]. A phylogenetic tree was constructed based on four plastid sequences: *trnL *intron*, trnL-F *IGS, *rpl16*, and *accD-psa1* IGS and one nuclear repeated sequence: rDNA ITS [13]. The tree contains valuable information but also many unsolved relationships concerning the evolution of the genus and the speciation process, especially in Madagascar. Several approaches using TE could be used to solve this problem such as SSAP [14] and REMAP [15]. Both approaches enable estimation of the genome-wide TE distribution. A third experimental procedure reveals site-specific insertion polymorphism [16]. This method requires the identification of information on flanking sequences to facilitate the design of primers to detect polymorphism by PCR. Sequencing of a *C. canephora* BAC clone (46C02, accession no. EU164537) enabled identification of a new MITE, named *Alex-1* (Figure 1), in the 12th intron of a gene (*g3*) of this BAC clone [17]. 

In this paper, we characterize this novel MITE family in the *C. canephora* genome. We also report the results of a study on the insertion polymorphism of the MITE *Alex-1* at the *g3* locus using PCR approaches on a representative set of *Coffea* species and a representative set of *C. canephora* diversity groups [18–21]. Our results revealed high insertion polymorphism of the *Alex* MITE at the *g3* locus, which was not linked to the phylogenetic relationships of the *Coffea* species studied here. Taken together, these results suggest greater gene flow between species than previously thought. 

## 2. Material and Methods

### 2.1. Plant Material

Twenty-eight *Coffea* species grown in tropical greenhouses at the IRD center in Montpellier (France) were used in this study. They represent the natural ecogeographical distribution of the genus. One plant from a related genus, *Psilanthus*, was included in the survey. One to 11 plants (genotypes) per species were analyzed (depending on the number of samples available in the collection) except for *C. canephora* for which 71 accessions were included (see Supporting Material available at doi: 10.4061/2011/3584122). For the latter species, DNAs from 12 plants from the Ugandan diversity group were kindly provided by P. Musoli from NARO/COREC (Uganda) and T. Leroy from CIRAD (France).

### 2.2. DNA Isolation

DNA was extracted and purified using Qiagen DNeasy mini Kits (Hilden, Germany) according to the manufacturer's instructions. DNA quantification was performed on a NanoDrop TM 1000 Spectrophotometer (LabTech, France).

### 2.3. Identification of the MITE *Alex*

*De novo* identification of the MITE was performed using dot-plot alignments (DOTTER software, [22]) based on the presence of inverted repeats at both ends and target site duplication. Evaluation of the redundancy of the MITE in nucleotide sequences was conducted by BLAST searches using MITE *Alex* as a query against public databases of *C. canephora* (56,231) Expressed Sequence Tags (EST) (with an *e*-value cut-off of 10*e* ^−4^).

### 2.4. PCR Amplifications

Two primers were designed in conserved regions according to the 46C02 BAC sequence (accession no. EU164537) to check the presence/absence of the MITE *Alex-1* at the *g3* locus in the different accessions used in this study. The forward primer was designed in the 11th exon of that gene and the reverse primer in the 13th exon (see Supporting Material 1). The primer sequences used were: G3F: 5′ GTT-TGG-TTG-CTG-GGT-CTC-AT 3′ and G3R: 5′ CGA-CAA-GAG-GAA-AGC-CTC-AC 3′. The expected amplicon is 1093 bp long when the MITE *Alex-1* is present and 916 bp when it is absent (see supporting Material 1).

The PCR conditions were 94°C for 1 min. followed by 35 cycles at 94°C for 1 min, 58°C for 30 sec, 72°C for 45 sec, and a final elongation period at 72°C for 4 min. PCR products were observed by electrophoresis in 1% agarose gel after staining with ethidium bromide.

### 2.5. PCR Product Sequencing

In order to check the absence of insertion—as oppose to excision—of a former inserted MITE *Alex-1* at the *g3* locus, several PCR products from different species were sequenced. After electrophoresis, the bands at 1093 or 916 bp were excised from the gel using a razor blade. DNA was purified using a Quiagen PCR purification kit according to the manufacturer's recommendations and sent for sequencing to Eurofins-MWG (Ebersberg, Germany). Sequences were aligned using ClustalW software.

## 3. Results

### 3.1. Identification of Alex, a Novel MITE Family, in *C. canephora*

A BAC clone at the *CcEIN4* locus in *C. canephora *(BAC clone 46C02, accession no. EU164537) was recently sequenced [17]. This represented the first complete BAC clone ever sequenced in the *Coffea* genus. The typical structural features of a Miniature Inverted-repeat Transposable Element (MITE) were detected in the 12th intron of the *g3 *gene, encoding a putative protein (nucleotides 12468…12645 of the BAC clone). This element, named *Alex-1*, was flanked by the 3 bp direct repeat AGT, generated upon the insertion of the element (Target Site Duplication, TIR) and had 18 bp Terminal Inverted Repeats (TIR) at both ends of the element. The small sequence size (178 bp), rich A/T composition (73.4%), and the ability to form secondary structures characterized *Alex-1 *(Figure 1).

To further characterize this element, the nucleotide sequence of *Alex-1* was compared with the public nucleotide sequences of *C. canephora* comprising 56,231 Expressed Sequence Tags. BLAST searches produced 42 significant hits, suggesting that *Alex-1* belongs to a large family of MITE elements frequently associated with transcribed sequences. A BLASTN search performed on nonredundant (nr) public libraries did not produce any significant hit (length, percentage, identity, and *e*-value), except in *Coffea* genomic sequences.

### 3.2. Analysis of Genomic Polymorphism Associated with the MITE *Alex-1* at the *g3* Locus

Table 1(a) shows the presence or absence of the *Alex-1* MITE at the *g3* locus in the different plants analyzed. Within-species polymorphism in *C. canephora* is presented in Table 1(b) (see Supporting Material 3). 

The majority of the species (18/28) displayed total absence of the MITE at the *g3* locus, whereas 7/28 were homozygous for its presence and 3/28 displayed heterogeneous patterns. Only *C. liberica* var. *devewrei* and *C. canephora * displayed all three genotypes, homozygous +/+, homozygous −/−, and heterozygous +/−.

Two species or taxons showed a majority of homozygous genotypes, +/+ (*C. sp* N'Koumbala) or −/− (*C. humilis*), few heterozygotes +/−, but not the reciprocal homozygous, −/− (*C. sp* N'Koumbala) or +/+ (*C. humilis*).

**Figure 2:**
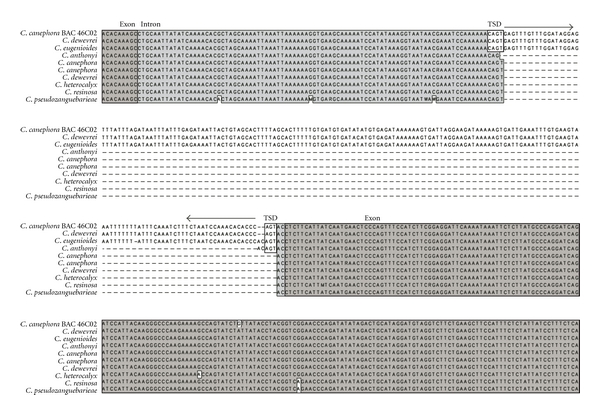
Sequence alignment of *Alex-1* insertion site in different *Coffea* species. *C. anthonyi* is the only negative sequence displaying the presence of a Target Site Duplication (TSD) (empty boxes in the figure). Black arrows indicate the presence of the Terminal Inverted Repeats (TIRs).

At the sequence level, a closer look at the locus of insertion for the presence of* Alex-1* in both positive and negative plants showed that only one of the negative individuals, *C. anthonyi*, displayed the remnant of a TSD (Target Site Duplication) sequence that indicates the former presence of a MITE and thus its transposition (Figure 2). This process of excision was precise since the whole element was removed from the site of insertion, and no large deletion occurred in the flanking regions. 

Interestingly, in the African species, no link was found between the geographical origin of the species and the presence of the *Alex-1* MITE. Indeed, *Alex-1* was present in East, West and Central African species (Mozambicoffea and Eucoffea, resp.). However, all the Mascarocoffea species originating from islands in the Indian Ocean (Madagascar, Mauritius, and Comoros) lacked the MITE at the *g3* locus. Most *Coffea* species appear to be homozygous since only seven plants out of 129 were heterozygous.

The situation of *C. canephora* is particularly interesting since the only diversity group E, originating from the Congo/Cameroon region, contains homozygous −/− genotypes (Table 1(b) and see Supporting Material 4). The heterozygotes detected in groups D, A, and C were previously identified as being intergroup hybrids all with a group E genotype in their pedigree [19]. Similarly, heterozygotes in group E turned out to be hybrids between group D and group E genitors [19]. The homozygous individual in group E was collected in RCI (Ivory Coast), far from the place of origin of that diversity group, which is in the Congo/Cameroon region. This particular plant was certainly introduced into RCI for improvement purposes quite a long time ago and has certainly undergone several crosses and backcrosses leading to an introgressed form bearing the inserted locus on both homologous chromosomes (+/+) (Accession 319, see Supporting Material 2 and 4). The diversity group E thus appears to be the only one among the *C. canephora* groups to be characterized by the absence of *Alex-1* at the *g3* locus.

**Figure 3:**
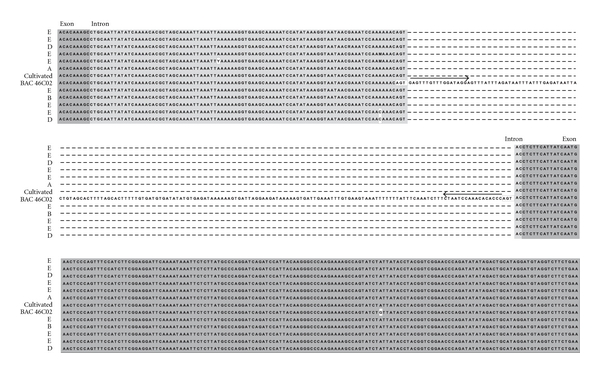
Sequence alignment of *C. canephora* accessions displaying the absence of *Alex-1* compared with BAC 46C02 sequence. The absence of a TSD indicates that the MITE was not excised from this site and that insertion never occurred. The accessions sequences were 739, 504, 738, 665, 663, 730, 321, 604, 345, 651, and 725 for wild accessions belonging to A, B, D, and E genetic diversity groups, and to the cultivated BD55 accession from Cameroon. Empty boxes: TSD. Black arrows: TIRs.

Analysis of the sequences in seven negative genotypes in the E diversity group and in negative genotypes in other diversity groups revealed no remnant TSD sequences, suggesting that the absence of *Alex-1* at the *g3* locus is more likely due to a lack of insertion than to postdifferentiation excision (Figure 3).

The closest relative to the genus *Coffea*, a plant from the *Psilanthus* genus, also lacked the insertion of the *Alex-1* MITE at the *g3* locus.

## 4. Discussion

In this paper we describe the first MITE characterized in the genome of a *Coffea* species that has no homologs in other published sequences, it appears thus as specific to the *Coffea* genus. We studied the insertion polymorphism of this MITE at the *g3* locus. The distribution pattern of *Alex-1* at this locus in the *Coffea* and *Psilanthus* species strongly suggests that insertion occurred early in relation with the evolution of these genera, although certainly after the divergence between *Coffea* and *Psilanthus* as *Alex-1* was not found in *Psilanthus*. However, not enough *Psilanthus* species or genotypes were analyzed to confirm this hypothesis. 

**Figure 4:**
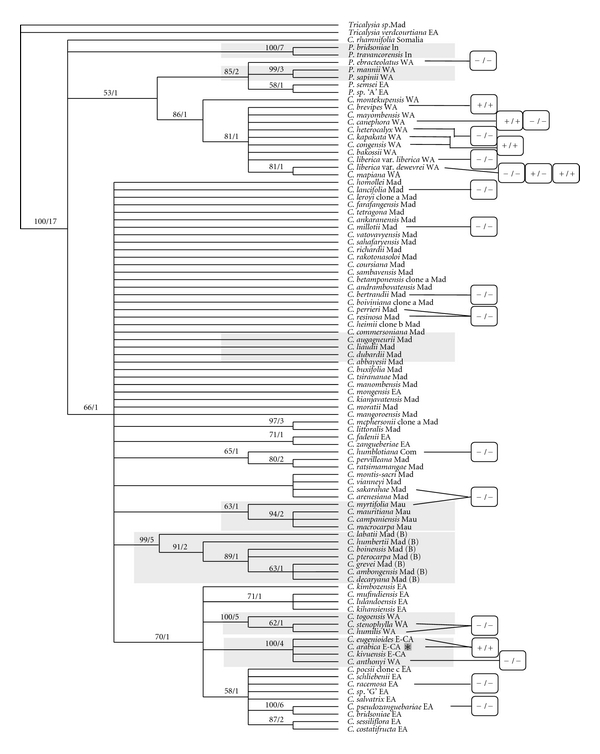
Insertion polymorphism of the MITE *Alex-1* superimposed to a phylogenetic tree established by Maurin et al. [13]. Except for the species from the Indian Ocean islands, which are homozygous −/−, all possible allelic associations are found on the African continent despite the geographical region, West or East. *: *C. arabica* is the sole tetraploid *Coffea* species. WA: West and Central Africa, Mad: Madagascar, Com: Comoros, Mau: Mauritius, EA: East Africa. All the species analyzed in the present study are not represented on the tree and reciprocally.

Because the distribution of the MITE *Alex-1* does not corroborate previous phylogenetic studies [13] and was found in *Coffea* species independently of their eco-geographical distribution (Figure 4), its insertion at the *g3* locus most probably occurred before the spread of the genus in Africa but probably after the colonization of Madagascar and the other islands in the Indian Ocean by one or several ancestral *Coffea* species if we consider the hypothesis that the genus originated in the African continent.

Its insertion certainly occurred before the formation of the *C. arabica* species, which is the only allotetraploid in the *Coffea* genus, originating from Southern Ethiopia and most probably resulting from a cross between *C. eugenioides* and *C. canephora * [23] both being only or mostly homozygous +/+. 

When the insertion of a transposable element occurs, it is always in heterozygous form. The probability that the same TE is inserted at the same locus, at exactly the same spot on both homologous chromosomes, is almost nil. If the insertion does not modify a gene function leading to an advantage or disadvantage in terms of selection, its maintenance in the genome responds to a neutral model and may be conserved or eliminated in the following generations. In the present case, as no link was found between the presence or absence of *Alex-1* at the *g3* locus and the habitat type of the species, the neutral situation probably applies. It is still not clear why species then became preferentially fixed for the presence or absence of the TE, if this was not merely random.

Four species displayed the presence of heterozygous genotypes and only two (*C. canephora* and *C. liberica* var *dewevrei*) showed the three possible patterns (Table 1(a)), homozygous (+/+ and −/−) and heterozygous (+/−). It is very likely that because of the size of the sample, all possible situations have not yet been identified in all the species. It is also possible that some fixation and/or divergence events are actually still underway. The most intriguing example is *C. liberica* var *dewevrei*, which displays the three genotypes (homozygous and heterozygous), while *C. liberica* var *liberica* is fixed for the absence of *Alex-1* (homozygotes −/−) and *C. liberica* var *koto* is fixed for the presence of *Alex-1* (homozygotes +/+). *C. liberica* var *dewevrei* may still be in the fixation process but this could take quite a long time, as the three genotypes are encountered with equal frequency.

In the cases of *C. sp* N'Koumbala and *C. humilis*, no −/− or +/+ homozygotes were identified (Table 1(a)), which does not mean that these types of homozygotes do not exist but simply that they were not present in the sample we analyzed. The presence of heterozygotes (+/−) can result from an allelic equilibrium with a low frequency of positive alleles in *C. sp* N'Koumbala and of a negative allele in *C. humilis,* but in such a situation, the reciprocal homozygote would also be expected to be present, and this was not the case in our sample. Another possible explanation for this low allelic frequency is that interspecific crosses, even if very rare, may happen throughout the Cameroon/Congo region and in RCI, which are hot spots of diversification and secondary centers of speciation for *Coffea* species [24]. 

The *C. canephora* group E insertion pattern suggests possible interspecific hybridization and gene flow. Indeed, it is the only group in this species that lacked *Alex-1* at the *g3* locus. The absence of TSD in the sequenced amplicons (Figure 3) indicates that *Alex-1* has never been inserted at that locus, and consequently, that its absence is not the result of transposition to another site. All the other genotypes, whatever diversity group they belong to, are +/+ homozygotes, it is thus highly likely that the common ancestor of *C. canephora* underwent the insertion of *Alex-1* at the *g3* locus and then evolved towards the fixation of the insertion (+/+ pattern). Group E, and certainly other unidentified genomic sequences, could then result from an introgression following a cross with a neighboring (sympatric) species and backcrosses to recover *C. canephora* properties.

Interestingly, under this hypothesis of introgression, *C. sp* N'Koumbala is a possible candidate to be the provider of the absence of insertion if an allelic equilibrium remains in this species. Indeed, this taxon grows in the same region as plants from diversity group E, but additional comparative sequencing of the *g3* locus, including flanking regions, is necessary to confirm or reject the hypothesis.

It is also possible that this particular group derives from a sister plant to the plant that integrated the MITE, the latter resulted in the full *C. canephora* lineage except for group E. In this case, group E may derive from a population that lived in sympatry with *C. canephora*, from which it has never completely genetically separated due to cross hybridization. If this is the case, the genome region that contains the *Alex-1* MITE was preserved from recombination, which should have led to +/− and +/+ genotypes. However, genotypes that are found in artificial intraspecific hybrids make this hypothesis unlikely.

## 5. Conclusion

The insertion pattern of the *Alex-1* MITE at the *g3* locus in *Coffea* species indicates an original path of species differentiation including gene flows between ancestral forms that happened before the present. Recent collecting missions very occasionally identified natural interspecific hybrids or sympatric populations of *Coffea* species. However, it is known that such events can happen in the wild (*C. arabica* being the best example), or in displaced populations in functioning or abandoned coffee plantations [25]. Changing environmental conditions and habitat modification could certainly have led to cohabitation of two or more species in limited areas where their specific phenology was disturbed, thus allowing cross pollination. Subsequent environmental changes could have led to the expansion of favorable habitats, resulting in the isolation of the newly formed species.

MITEs thus appear to be a powerful tool to analyze these speciation events and to trace the phylogenetic relationships between species and if the number of specific insertion sites is sufficient to enable the establishment of an event chronology [8].

## Figures and Tables

**Table 1a:** (a) Insertion polymorphism of the *Alex-1* MITE at the g3 locus among a representative set of *Coffea* species and a close relative *Psilanthus ebracteolatus. *

Species analyzed	No. of individuals	+/+	−/−	+/−	Origin
*C. arabica*	3	3	0	0	E. Africa, Ethiopia
*C. eugenioides *	9	9	0	0	E. Africa Kenya
*C. pseudozanguebariae*	11	0	11	0	E. Africa Kenya
*C. racemosa*	11	0	11	0	E. Africa Tanzania
*C. liberica* var *liberica *	10	0	10	0	W. Africa RCI
*C. stenophylla*	10	0	10	0	W. Africa RCI
*C. humilis*	10	0	8	2	W. Africa RCI
*C. canephora*	71	53	8	10	W. and C. Africa
*C. congensis*	5	5	0	0	C. Africa RCA
*C. liberica *var.* dewevrei *	10	4	3	3	C. Africa RCA
*C. liberica *var.* koto *	3	3	0	0	C. Africa Cameroon
*C. brevipes*	10	10	0	0	C. Africa Cameroon
*C. heterocalyx*	1	0	1	0	C. Africa Cameroon
*C. anthonyi*	7	0	7	0	C. Africa Cameroon
*C. sp *N'Koumbala	10	8	0	2	C. Africa Cameroon
*C. sp *Mayombé	3	3	0	0	C. Africa Congo R.
*C. kapakata*	2	2	0	0	C. Africa Angola
*C. myrtifolia*	3	0	3	0	Mauritius
*C. resinosa*	1	0	1	0	Madagascar
*C. tsirananae*	1	0	1	0	Madagascar
*C. lancifolia*	1	0	1	0	Madagascar
*C. perrieri*	1	0	1	0	Madagascar
*C. sakarahae*	1	0	1	0	Madagascar
*C. millotii*	1	0	1	0	Madagascar
*C. dolichophyla*	1	0	1	0	Madagascar
*C. heimii*	1	0	1	0	Madagascar
*C. bertrandii*	1	0	1	0	Madagascar
*C. humblotiana*	1	0	1	0	Comoros
*P. ebracteolatus*	1	0	1	0	W. Africa RCI

Total	200	100	83	17	

E. Africa: East Africa; W. Africa: West Africa; C. Africa: Central Africa.

RCI: République de Côte d'Ivoire (Ivory Coast); RCA: République Centre Africaine (Central African Republic).

**Table 1b:** (b) Insertion polymorphism of the *Alex-1* MITE at the *g3* locus among a representative set of the diversity groups of the *Coffea canephora* species as defined by Gomez et al. [19].

Diversity group	No. of individuals	**+/+**	−/−	**+/**−	Origin
D	29	28	0	1* (D**E**A)	Guinea/RCI
A	2	1	0	1* (A**E**)	Cameroon/Congo
B	3	3	0	0	RCA
C	9	8	0	1* (C**E**)	Cameroon/Congo/RCA
E	16	1	8	7* (**D**E)	Congo/Cameroon/RCA
O	12	12	0	0	Uganda

Total	71	53	8	10	

+/+ & −/−: Homozygote for presence and absence, respectively; +/−: Heterozygote. *: intraspecific hybrids.
